# Rye polyphenols and the metabolism of n-3 fatty acids in rats: a dose dependent fatty fish-like effect

**DOI:** 10.1038/srep40162

**Published:** 2017-01-10

**Authors:** Fayçal Ounnas, Michel de Lorgeril, Patricia Salen, François Laporte, Luca Calani, Pedro Mena, Furio Brighenti, Daniele Del Rio, Christine Demeilliers

**Affiliations:** 1 Laboratoire TIMC-IMAG CNRS UMR 5525, Cœur et Nutrition, Université Grenoble-Alpes, Grenoble, France; 2 Laboratory of Fundamental and Applied Bioenergetics, Environmental and Systems Biology, Inserm, U1055, Université Grenoble-Alpes, Grenoble France; 3 Département de Biochimie, Pharmacologie et Toxicologie, Unité Biochimie Hormonale et Nutritionnelle, Centre Hospitalier et Universitaire de Grenoble, France; 4 Laboratory of Phytochemicals in Physiology, Department of Food Science, University of Parma, Medical School, Building C, Parma, Italy

## Abstract

As long-chain fatty acids (LCFA) of the n-3 series are critically important for human health, fish consumption has considerably increased in recent decades, resulting in overfishing to respond to the worldwide demand, to an extent that is not sustainable for consumers’ health, fisheries economy, and marine ecology. In a recent study, it has been shown that whole rye (WR) consumption improves blood and liver n-3 LCFA levels and gut microbiota composition in rats compared to refined rye. The present work demonstrates that specific colonic polyphenol metabolites may dose dependently stimulate the synthesis of n-3 LCFA, possibly through their microbial and hepatic metabolites in rats. The intake of plant n-3 alpha-linolenic acid and WR results in a sort of *fatty fish-like effect*, demonstrating that the n-3 LCFA levels in blood and tissues could be increased without eating marine foods, and therefore without promoting unsustainable overfishing, and without damaging marine ecology.

Long-chain fatty acids (LCFA) of the n-3 series — also called marine LCFA because marine foods are the main source of these lipids — are critically important for human health^1^. Fish consumption has considerably increased in recent decades, resulting in overfishing to respond to the worldwide demand^2^, and, at the same time, sea and ocean pollution have also increased over the planet^3^. As a consequence, it is more and more difficult for consumers to obtain high-quality marine foods at reasonable prices^2^. The present situation is not sustainable for consumers’ health, fisheries’ economy, and marine ecology.

We need alternative sources of marine n-3 LCFA, and this made fish farming (or pisciculture) grow relevantly. However, farming carnivorous fish (e.g. salmon) does not reduce pressure on wild fisheries, since they require fishmeal extracted from wild forage fish. On the other hand, the amounts of marine n-3 LCFA in herbivorous fishes (e.g. carp and tilapia) are quite low and will not be able to meet the future growing demand.

Seaweed farming may be another solution, and marine n-3 LCFA have now been produced in large-scale cultivation of microalgae, but this is unlikely to become an affordable reality in the short-term, particularly because of high production costs, quite limited yields in marine n-3 LCFA, and consumer reluctance. Finally, marine n-3 LCFA could also be produced from terrestrial plants via transgenic means, which will eventually clear regulatory hurdles for commercialisation, but societal acceptance remains in question. For the present time, no affordable, environmentally sustainable, and socially acceptable solution exists.

Since humans have the ability to synthesize marine n-3 LCFA from plant n-3 alpha-linolenic acid (although insufficiently), one solution would be to stimulate the endogenous production of n-3 LCFA by the human body. For instance, certain polyphenols have been shown able to increase the concentrations of marine n-3 LCFA in animals^4^,^5^ and humans^6^,^7^. In a recent study, it has been shown that whole rye (WR) consumption improves blood and liver n-3 LCFA levels and gut microbiota composition in rats compared to refined rye^8^. This might be partly involved in the health benefits associated with WR consumption, particularly a lower risk of metabolic syndrome and cancer^9^,^10^.

However, the totality of mechanisms by which WR is beneficial is not fully understood. Also, the specific substances potentially implicated in WR health benefits — and present in the outer part of the rye grain — have not been fully identified. As an example, WR contains several polyphenols, including lignans and phenolic acids, which can influence the endogenous biosynthesis of n-3 LCFA. Rye lignans are converted by gut bacteria into enterodiol and enterolactone^11^,^12^ that possess hormonal activity, and this may partly modulate the synthesis of n-3 LCFA in humans^13^,^14^,^15^. In addition, WR consumption in rats results in increased urinary excretion of phenolic acid metabolites, which may also influence the metabolism of n-3 LCFA^5^,^8^,^16^. In the above-cited animal study comparing refined rye and WR, we did not evaluate the dose–effect relationship — a useful approach to demonstrate a causal effect — between rye polyphenols and n-3 LCFA, and whether specific rye polyphenol metabolites were involved in the metabolism n-3 LCFA^8^. Therefore, the present study was designed to confirm the effect of rye polyphenols on the endogenous metabolism of n-3 LCFA in rats, in order to examine a dose–effect relationship and to identify which polyphenol metabolites are potentially involved in the process. We compared three groups of rats, where two groups were fed two different doses of WR, whereas a control group did not consume rye. We used a multiple linear regression model to analyse the associations between urinary rye polyphenol metabolites and systemic and hepatic marine n-3 LCFA.

## Materials and Methods

### Animals and experimental diets

Thirty-six male Wistar rats (Charles River Laboratories, l′Arbresle, France, baseline body weight 75–100 g) were fed a standard diet (A04, SAFE Diets, France). They were housed in individual cages under conditions of constant temperature and humidity and a standard light-dark cycle (12 h/12 h). Tap water and standard diet were provided *ad libitum.*

The standard diet was A04, from SAFE Diets (France) and did not contain any WR (control diet, CT). The experimental diets were prepared by mixing the same standard diet A04 flour with either 39% WR (WR39) or 79% WR (WR79) as a replacement for A04 cereals. The amounts of nutrients provided by the two experimental diets were adjusted to the recommendations of the American Institute of Nutrition Rodent Diets-93^17^.

### Experimental design

The rats were cared for in accordance with the European Council Directive 86/609/EEC on the care and use of laboratory animals (OJL 358). Protocols were carried out under license from the French Ministry of Agriculture (Permit N° A380727) and approved by the Committee on the Ethics of Animal Experiments of the University of Grenoble (Permit N° 113_ LBFA-FO-01). All efforts were made to minimise animal suffering.

The animals were acclimatised one week before being randomly distributed into three groups (n = 12/group). The rats were then fed either the CT or the WR39 or the WR79 diets during 12 weeks. Weight and food consumption were recorded weekly. One week before sacrifice, urine was sampled in individual metabolic cages. At the end of the experiment, plasma and liver were sampled. All samples were immediately frozen in liquid nitrogen and stored at −80 °C until analysis.

### Analysis of polyphenolic compounds in plasma and urine

Urine samples were diluted with 0.1% aqueous formic acid and filtered through a 0.45 μm nylon filter prior to UHPLC-MS^n^ (LTQ XL, Thermo Fisher Scientific Inc., San Jose, CA, USA) as previously reported^8^.

### Fatty acid analyses

Plasma and liver lipids were extracted in hexane/isopropanol and quantitated as previously described^6^,^7^. Briefly, methylated fatty acids were extracted with hexane, separated, and quantified by GC using a 6850 Series gas chromatograph system (Agilent Technologies, Palo Alto, CA, USA). Plasma is the main biological factor involved in fatty acid distribution in the body and the liver is regarded the main source of endogenous synthesis of polyunsaturated fatty acids in mammals. We focused our analyses on the changes in n-6 and n-3 fatty acids, in particular eicosapentanoic acid (EPA, 20:5n-3) and docosahexanoic acid (DHA, 22:6n-3).

### Statistical analysis

Between-group differences in physiological characteristics, urinary polyphenols and blood and liver fatty acids were evaluated using analysis of variance, and individual group differences were evaluated when necessary with post-hoc Fisher’s LSD test (significance *p* < 0.05) using the Minitab V17 software (GrimmerSoft, Paris, France) and SPSS Version 20.0 (SPSS Inc., Chicago, IL, USA). All the results are presented as mean ± SD.

The association between urinary polyphenol metabolites and plasma n-3 LCFA was evaluated by using multiple linear regression model where the levels of fatty acids obtained in plasma (%) were the predicted variable and the polyphenols metabolites excreted in urine (μmol/L) were the predictor variables according to the following model (using the Minitab V17 software): Yij = Gi + a Pij_1_ + b Pij_2_ + c Pij_3_ + …+ x Pij_16_, where Gi was the diet effect (i = CT diet or WR 39% diet or WR 79% diet) and Pij the effect of polyphenol metabolite j (j = 1, 2, 3… or 16); Yij being the fatty acid response to the changes in i (diets) and j (polyphenol metabolites). Because of the high correlation between blood and liver n-3 LCFA [r = 0.87 for DHA and r = 0.72 for EPA], analyses were limited to the association between urinary polyphenols and plasma n-3 LCFA.

## Results

### Macronutrient and fatty acid composition in the three groups

Macronutrients in the three groups were similar (Table 1). Dietary fatty acids in the three groups did not differ, and, in particular, there was no difference in the amounts of the essential alpha-linolenic (18:3n-3) fatty acids. As shown on Table 1, the small increase (as % of total fatty acids) in the WR79 and WR39 groups, compared with the control group, was compensated by a decrease in total fat intake.

### Body weight and total food consumption

Mean body weight (437 ± 32 g, 418 ± 31 g, and 422 ± 32 g, respectively, in the control, WR39, and WR79 groups) and mean food consumption (26.4 ± 1.9 g/day, 25.9 ± 2.3 g/day, and 25.0 ± 2.0 g/day, respectively) after 12 weeks were not statistically different among the three groups (*p* > 0.05).

### Urinary excretion of polyphenol metabolites

Many polyphenol metabolites were identified in the urine of rats fed the three diets (Table 2). The level of each metabolite excreted in urine increased with the amounts of WR ingested by rats. The WR-fed rats excreted significantly more enterolactone and enterolactone-glucuronide as well as several phenolic acid metabolites (such as sinapic and ferulic acid glucuronides, and ferulic acid, dihydroferulic acid, and caffeic sulphates) compared with controls, and the WR79 rats excreted significantly more metabolites than the WR39 rats. The amounts of each polyphenol metabolite in urine increased linearly with the amounts of WR ingested.

### Plasma and liver fatty acids

Regarding polyunsaturated fatty acids in plasma (Table 3) and liver (Table 4), n-3 LCFA (in particular, EPA and DHA) increased significantly with the amounts of WR ingested. At the same time, saturated and monounsaturated fatty acids did not change significantly (data not shown), whereas certain n-6 fatty acids (in particular, 18:2n-6 and 20:4n-6 in plasma and 18:2n-6 in liver) decreased significantly with the amounts of WR ingested (Table 3 and Table 4).

### Polyphenols and LCFA relationships

16 polyphenol metabolites were identified and quantified in urine, and their associations with plasma levels of EPA and DHA were investigated. Although unexpected, significant changes in 18:2n-6 in both plasma and liver were observed. For this reason, the relations between urinary polyphenol excretion and 18:2n-6 were also investigated. Polyphenol metabolites were sequentially tested and deleted from the model when there was no significant association. The final models integrated the effects of the experimental diets (as a major cofactor), and specific polyphenol metabolites were shown to be associated with EPA, DHA, or 18:2n-6. Three models were thus described, displaying the highest significant associations between the identified urinary polyphenol metabolites and plasma levels of EPA, DHA, and 18:2n-6. In particular, hydroxyphenylpropionic acid-*O*-sulphate was significantly associated with DHA (r^2^ = 0.39, *p* < 0.001) and 18:2n-6 (r^2^ = 0.36, *p* < 0.01), whereas 3-(3′-hydroxyphenyl)propionic acid was significantly associated with EPA levels (r^2^ = 0.39, *p* < 0.001). We found no significant association between lignan metabolites (enterolactones) and either EPA, DHA, or 18:2n-6. When considering the final models, 64% of the variability of 18:2n-6 was explained by the experimental diet and hydroxyphenylpropionic acid-*O*-sulphate, 59% of the variability of EPA was explained by the experimental diet and 3-(3′-hydroxyphenyl) propionic acid, and 69% of the variability of DHA was explained by the experimental diet, hippuric acid, benzoic acid-*O*-sulphate, and hydroxyphenylpropionic acid-*O*-sulphate (all *p* < 0.01).

## Discussion

### Summary

The health effects of n-3 LCFA are well established^1^,^18^. There are two main sources of n-3 LCFA in humans (and most animals, including rats): one is exogenous — i.e. marine foods, essentially fatty fish — while the other is endogenous synthesis, starting with the desaturation and then elongation of the *essential* plant n-3 alpha-linolenic acid^14^. The synthesis of EPA and DHA is partly hormone-dependent^13^,^15^. In rats and humans, the synthesis of n-3 LCFA from alpha-linolenic acid is thought to be insufficient to satisfy individual needs, especially during pregnancy. Thus, we must eat fatty fish — or n-3 LCFA supplements — to get sufficient amounts of *marine* n-3 LCFA, as these lipids are critical for optimal development and function of the brain and heart^19^. The environmental crisis, however, particularly overfishing and sea pollution, raises the question of whether it is really possible to ensure the *marine* n-3 LCFA needs of the entire population at acceptable costs^2^,^3^. The answer is clearly negative, so alternative sources of n-3 LCFA must be urgently identified. We have proposed (already in 2008) that one possibility would be to stimulate the endogenous synthesis of *marine* n-3 LCFA from their plant precursor alpha-linolenic acid, which can be consumed in much larger amounts in our western diets^6^,^7^. It was shown that some polyphenols could stimulate the endogenous synthesis of *marine* n-3 LCFA in rats and humans^4^,^5^,^6^,^7^,^8^, and, in particular, that polyphenols of WR seem very active by stimulating the different steps of n-3 LCFA synthesis from alpha-linolenic acid to EPA, from EPA to 22:5n-3, and from 22:5n-3 to DHA^8^.

The main aim of the present study was then to examine whether WR consumption may increase levels of n-3 LCFA in the blood and liver of rats in a dose-dependent manner. We show a dose–effect relationship between WR consumption and both EPA and DHA in blood and liver, paralleled with significant associations between urinary polyphenol metabolites and blood levels of EPA and DHA. We finally identify specific rye polyphenol metabolites associated with the specific effect on EPA, DHA, and 18:2n-6. The polyphenols present in rye potentially involved in the regulation of EPA and DHA (and 18:2n-6) are not lignan metabolites — known to have a hormonal effect — but phenolic acid derivatives whose origin is partly influenced by the gut microbiota. On the other hand, we have shown in a previous study that WR consumption influenced gut microbial profiles in rats^8^. Further studies will have to investigate whether the WR-induced changes in the gut microbiota, and the subsequent transformation of rye polyphenols in more active forms, are the primary causes of the changes in liver and blood n-3 LCFA levels. Studies are also needed to examine which specific bacterial species are involved in the transformation of rye polyphenols.

### Whole rye and polyphenol metabolism

In this study, incorporation of WR (39 and 79%) in the diets resulted in highly significant changes in the urinary excretion of polyphenol metabolites. For most of them, doubling the intake of WR resulted in at least a double concentration of polyphenol metabolites in urine. Main urinary metabolites were phenolic acid derivatives, particularly hippuric acid and glucuronide and sulphate conjugates of ferulic acid, sinapic acid, and hydroxyphenylpropionic acid, as also shown in a previous study^8^. Lignan metabolites found in urines were enterolactone and enterolactone-glucuronide. The higher excretion of phenolic acid derivatives in comparison with lignan metabolites was expected since phenolic acids, in particular ferulic acid oligomers, are the main components of the phenolic fraction of whole rye^8^.

We cannot exclude the possibility that the high amounts of WR in the two experimental groups had some effects on the gut microbiota with, for instance, modification of rye lignan metabolism. Phenolic acids might also inhibit certain species or families of the gut microbiota involved in the bioactivation of rye lignans. Further studies are needed to investigate this issue. Also, we cannot exclude the possibility that we missed some lignan metabolites when analysing the complex spectrum of polyphenols in rat urine.

### Whole rye and n-3 LCFA

The present study confirms and reinforces our previous findings regarding the effect of WR polyphenols on blood and liver n-3 LCFA^8^. We show that higher amounts of dietary polyphenols are associated to higher EPA and DHA, suggesting a dose–effect relationship. Also, we show that the effect of rye polyphenols on EPA and DHA is observed in both liver and plasma, confirming the observations made in our previous study^8^. This suggests that at least one of the probable mechanisms by which rye polyphenols increase EPA and DHA levels is through stimulation of their synthesis in the liver, which is central in the overall production of EPA and DHA in the body^13^,^14^. Additionally, the level of the precursor in the n-3 synthetic chain (18:3n-3 or alpha-linolenic acid) was reduced in the liver of the rats receiving WR79, suggesting that it was possibly used as a substrate to produce EPA and then DHA. As 22:5n-3 — the intermediate compound between EPA and DHA — was also increased in both liver and plasma, it suggests that the whole n-3 LCFA pathway was stimulated by rye polyphenols.

### Rye polyphenols and n-3 LCFA

The next question is which rye polyphenol metabolite is directly involved in modulating the metabolism of n-3 LCFA. To investigate this issue, one experimental strategy would have been to treat rats with single polyphenols and measure the changes in n-3 LCFA in response to a challenge test. However, it is reasonable to hypothesise that individual phenolics fed to the animals would not exert the same effect, as a sort of adaptation and modulation of the gut microbiota has to be achieved through non-digestible components which are inevitably provided with wholemeal cereals, very likely corresponding the complex carbohydrates and fibres present in the outer part of the rye grain^8^,^20^,^21^. We have previously demonstrated that WR was able to modulate the microbiota composition inducing a stimulation of the n-3 LCFA synthesis^8^, and that several types of polyphenols — including anthocyanins present in black corn^4^ and phenolic acids present in wheat aleurone^5^ — are possibly active factors in the metabolism of n-3 LCFA in rats.

The alternative experimental strategy has been to feed rats with WR and to analyse the relations between n-3 LCFA levels and urinary metabolites of rye phenolics. In humans with metabolic syndrome, rye bread consumption was associated with increased EPA and DHA levels in plasma^22^. Thus, not surprisingly, we found strong correlations between specific polyphenol metabolites and n-3 LCFA levels. Because the synthesis of EPA and DHA is thought to be partly hormone-dependent^13^,^14^,^15^, we expected that rye polyphenols with hormonal activity — in particular the phytoestrogenic metabolites of lignans — would represent the main factors, but this was not the case, as we found no association between urinary enterolactones and blood n-3 LCFA. The main phenolic metabolites identified as factors in the interactions between rye polyphenols and n-3 LCFA are two phenolic acid derivatives produced by the gut microbiota, namely hydroxyphenylpropionic acid-*O*-sulphate (significantly associated with DHA) and 3-(3′-hydroxyphenyl)propionic acid (significantly associated with EPA). These specific metabolites are known to derive from the hydroxycinnamic acids contained in the bran^23^.

Given the known complexity of the metabolism of polyunsaturated fatty acids, which is influenced by many (generally unknown) dietary, hormonal, and metabolic factors, our data demonstrate how previously unexplored compounds and mechanisms must be taken into account and investigated in detail.

### Rye polyphenols and 18:2n-6 (linoleic acid)

We also found a strong negative association between plasma 18:2n-6 (linoleic acid) and hydroxyphenylpropionic acid-*O*-sulphate, a metabolite known to originate from microbial degradation and further phase II conjugation by enterocytes/hepatocytes of hydroxycinnamates such as ferulic acid derivatives^23^, main whole rye phenolics^8^. A negative effect of WR on liver and blood 18:2n-6 was not unexpected, as similar results were observed in a previous study^8^. This might is, however, a major finding, because 18:2n-6 is the precursor of arachidonic acid, whose biological effects are opposed to those of n-3 LCFA^24^. In brief, high levels of n-6 LCFA tend to be pro-inflammatory and pro-thrombotic — favouring cardiovascular disease — whereas higher levels of n-3 LCFA are anti-inflammatory and anti-thrombotic. The n-3 and n-6 LCFA are in competition at the level of several major enzymatic desaturation-elongation systems, as well as the prostaglandin and lipoxygenase pathways. It is thought to be the ratio n-3/n-6 rather than the absolute levels of n-3 and n-6 LCFA describing better the risk of cardiovascular disease^25^,^26^,^27^. If our data on 18:2n-6 are confirmed in humans, we may have identified a major (double) mechanism by which rye polyphenols exert their health effects, namely by increasing n-3 and by decreasing n-6 LCFA *in vivo*. To be noted is that arachidonic acid (20:4n-6) was significantly lower in the blood of the two WR groups in parallel with the respective blood levels of the precursor linoleic acid (18:2n-6). In contrast, liver arachidonic acid levels were not different in the three groups. This suggests that at liver level, rye polyphenols (or some other unidentified factors) increased the synthesis of arachidonic acid, possibly stimulating the whole n-6 pathway. This is not surprising because the involved enzymatic systems are similar for the n-3 and n-6 pathways.

Since 18:2n-6 is an *essential* fatty acid, whose unique sources are foods, the potential mechanism(s) by which rye polyphenols interfere with liver and blood 18:2n-6 levels may be different from the mechanism(s) by which they interfere with n-3 LCFA metabolism, i.e. desaturation and elongation of the *essential* precursor 18:3n-3. One possibility would be, given the differences in the 18:2n-6 content of the pellets (Table 1) and the small differences between groups in food consumption, that the overall intake in 18:2n-6 was smaller in the two WR groups and that after 12 weeks a difference between the three groups could be observed in blood and liver 18:2n-6 levels. However, this is unlikely to explain the large differences among the groups. Another possibility is that rye polyphenols may prevent, or reduce, the intestinal bioavailability of 18:2n-6, in the same way tea polyphenols have been reported to do, by decreasing fat absorption and increasing faecal lipid excretion^28^. This hypothesis raises two questions: first, why should absorption of 18:2n-6 and not that of other polyunsaturated fatty acids, particularly 18:3n-3 alpha-linolenic acid be impacted by rye polyphenols? Second, why should only one rye polyphenol metabolite — hydroxyphenylpropionic acid-*O*-sulphate — be involved in the inhibition of absorption of 18:2n-6? To understand the interactions between rye polyphenols and 18:2n-6, it would be critical to answer these two questions in future studies.

## Conclusion

This study confirms that certain polyphenols may stimulate the synthesis of n-3 LCFA, but demonstrates for the first time that this effect is linked to the transformation of specific polyphenols into their microbial and hepatic metabolites. Moreover, this work reports for the first time a dose-dependent effect of rye polyphenols on the synthesis of n-3 LCFA. In particular, if results on 18:2n-6 are confirmed in humans, we may have identified a major (double) mechanism by which rye polyphenols may exert their health effects, namely by increasing n-3 and by decreasing n-6 LCFA *in vivo*, a mechanism that had not been reported before. Of course these results should not be limited solely to rye phenolics, as the metabolites likely exerting the observed effects on essential fatty acid synthesis are common with other dietary sources. Nevertheless, the effects exerted by rye are remarkable and it might be hypothesised that indigestible carbohydrates provided with wholemeal rye may represent a contributing factor in shaping the gut microbiota of the animals, making it more prone to the production of specific polyphenols metabolites.

Further confirmation in humans of this work may therefore lead to a very promising conclusion. Eating plant n-3 alpha-linolenic acid and WR might result in a sort of *fatty fish-like effect*^4^,^5^,^6^,^7^,^8^, ideally proving that we can increase n-3 LCFA blood and tissue levels without eating marine foods, and therefore without promoting unsustainable overfishing, and without damaging marine ecology. This approach could also be advantageous as marine foods, especially fatty fish, often contain significant amounts of pollutants (e.g. pesticides and heavy metals) that might be toxic, especially for children, pregnant women and their foetuses, whereas n-3 LCFA are critically important for brain and eye development. A combination of the plant n-3 fatty acid and polyphenols (with an ‘active’ gut microbiota) might therefore represent a healthy alternative solution to the consumption of marine foods.

## Additional Information

**How to cite this article**: Ounnas, F. *et al*. Rye polyphenols and the metabolism of n-3 fatty acids in rats: a dose dependent fatty fish-like effect. *Sci. Rep.* **7**, 40162; doi: 10.1038/srep40162 (2017).

**Publisher's note:** Springer Nature remains neutral with regard to jurisdictional claims in published maps and institutional affiliations.

## Figures and Tables

**Table 1:** Macronutrient and fatty acid composition in the 3 groups.

	Control	WR39	WR79
Macronutrients (g/100 g pellet)			
Protein	15.7	15.1	13.5
Fat	2.8	2.4	2.0
Available carbohydrates	43.9	47	47.5
Fiber	16.8	15.9	16.6
Cellulose	3.6	3.3	2.9
Fatty acids (% of total fatty acids)			
C16:0	16.3	16.4	15.8
C18:0	2.6	1.7	1.4
C18:1n-9	20.6	20.2	19.8
C18:1n-7	1.2	1.5	1.8
C18:2n-6	51.6	51.1	49.7
C18:3n-3	4.2	5.0	6.4
Total SFA	19.7	19.0	18.2
Total MUFA	22.5	22.4	22.6
Total (n-3)	5.9	7.1	9.1
Total (n-6)	51.9	51.4	50.2
Polyunsaturated/saturated	2.9	3.1	3.3

SFA: total saturated fatty acids; MUFA: monounsaturated fatty acids.

**Table 2:** Urinary concentration of the main phenolic metabolites in the 3 groups.

Metabolite	Control	WR39	WR79
3-(3′-Hydroxyphenyl)propionic acid	25.85 ± 7.21	49.77 ± 15.29	67.56 ± 18.22
Hippuric acid	1040.67 ± 216.51	1740.24 ± 438.85	1719.92 ± 681.76
Enterolactone	nd	0.61 ± 0.25 b	1.37 ± 0.64 a
Benzoic acid-*O*-sulphate	21.65 ± 5.77 bc	29.71 ± 4.14 ab	42.42 ± 9.40 a
Coumaric acid-*O*-sulphate	29.34 ± 10.21	48.90 ± 16.21	75.94 ± 25.86
Phenylpropionic acid-*O*-sulphate	99.86 ± 26.61	135.09 ± 25.93	193.29 ± 43.38
Vanillic acid-4-*O*-sulphate	12.50 ± 4.84	16.80 ± 6.21	32.35 ± 9.68
Caffeic acid-*O*-sulphate	3.78 ± 1.51 bc	18.98 ± 6.97 ab	46.64 ± 18.06 a
Hydroxyphenylpropionic acid-*O*-sulphate	44.49 ± 9.67 b	114.49 ± 20.84 b	229.46 ± 54.51 a
Ferulic acid-4′-*O*-sulphate	80.68 ± 32.75 bc	149.32 ± 66.15 ab	405.23 ± 144.11 a
Dihydroferulic acid-4′-*O*-sulphate	4.71 ± 1.04 bc	7.96 ± 2.18 ab	14.76 ± 5.30 a
Sinapic acid-4′-*O*-sulphate	1.81 ± 0.78 b	4.72 ± 1.95 b	20.49 ± 6.93 a
Hydroxyphenylpropionic acid-*O*-glucuronide	7.66 ± 2.05 bc	16.24 ± 4.24 ab	26.57 ± 7.79 a
Ferulic acid-4′-*O*-glucuronide	4.66 ± 2.66	8.25 ± 3.67	19.30 ± 7.73
Sinapic acid-4′-*O*-glucuronide	10.37 ± 5.11 b	27.76 ± 12.18 b	74.23 ± 22.89 a
Enterolactone-*O*-glucuronide	0.82 ± 0.32 bc	2.29 ± 0.98 ab	5.53 ± 1.69 a

Data are expressed as median values (μmol/L ± SEM). “nd” means “not detected”. Different letters mean significantly different concentrations among the groups (*p* < 0.05).

**Table 3:** Plasma fatty acid in the 3 groups after 12 weeks of treatment.

	Standard diet Control			Whole rye diet 1 WR39			Whole rye diet 2 WR79			*P* value
	Mean	SD		Mean	SD		Mean	SD		
Fatty acids, % of total fatty acids										
C18:2n-6	22.09	1.68	a	19.88	2.36	b	17.08	1.79	c	***
C18:3n-6	0.36	0.08	ab	0.39	0.06	a	0.31	0.05	b	*
C20:2n-6	0.15	0.03	a	0.11	0.02	b	0.13	0.05	ab	*
C20:3n-6	0.49	0.10	b	0.48	0.11	b	0.64	0.13	a	***
C20:4n-6	26.21	5.07	a	21.59	4.16	ab	20.95	5.32	b	*
C22:4n-6	0.30	0.06	a	0.24	0.03	b	0.24	0.05	b	***
C18:3n3 (α-Linolenic)	0.83	0.15		0.88	0.18		0.81	0.14		
C18:4n3	0.05	0.02		0.07	0.02		0.05	0.03		
C20:5n-3 (EPA)	**1.51**	0.34	a	**1.81**	0.21	ab	**1.94**	0.39	b	***
C22:5n-3	**0.84**	0.19	b	**0.85**	0.14	b	**1.09**	0.22	a	***
C22:6n-3 (DHA)	**5.16**	0.39	b	**5.50**	0.52	b	**6.24**	0.52	a	***
TOTAL	100			100			100			
n3	8.40			9.11			10.13			
n6	49.59			42.68			39.34			
ratio (n-6/n-3)	5.9			4.7			3.9			

Values are means ± SD, n = 12 per group.

a, b, c: Mean values with different letters are significantly different(*p* > 0.05) one-way ANOVA.

*Indicate statistical difference between the groups: *p < 0.05, **p < 0.01, ***p < 0.001. n = 12 rats liver.

**Table 4:** Liver fatty acids after 12 weeks of treatment.

	Standard diet Control			Whole rye diet 1 Rye 39%			Whole rye diet 2 Rye 79%			*P* value
	Mean	SD		Mean	SD		Mean	SD		
Fatty acids, % of total fatty acids										
C18:2n-6	18.32	2.10	a	16.24	1.34	a	13.55	0.60	b	**
C18:3n-6	0.26	0.11		0.24	0.06		0.17	0.04		
C20:2n-6	0.19	0.04		0.16	0.02		0.18	0.06		
C20:3n-6	0.67	0.09	b	0.78	0.16	ab	0.93	0.22	a	*
C20:4n-6	21.15	0.85		20.98	1.42		20.75	1.44		
C22:4n-6	0.32	0.07		0.29	0.06		0.31	0.04		
C18:3n3 (α-Linolenic)	0.53	0.14	a	0.54	0.07	a	0.39	0.08	b	*
C18:4n3	0.05	0.03		0.05	0.01		0.03	0.01		
C20:5n-3 (EPA)	0.76	0.14	a	0.87	0.11	ab	0.92	0.18	b	*
C22:5n-3	0.94	0.17	a	0.96	0.13	a	1.23	0.17	b	*
C22:6n-3 (DHA)	6.88	0.78	a	8.29	0.76	b	9.06	0.49	b	**
TOTAL	100			100			100			
n3	9.14			10.72			11.64			
n6	40.92			38.69			35.88			
ratio (n-6/n-3)	4.48			3.61			3.08			

Values are means ± SD, n = 12 per group. a, b, c: Mean values with different letters are significantly different (*p* < 0.05).

*Indicate statistical difference between groups: *p < 0.05, **p < 0.001.
